# DEMOGRAPHIC CHARACTERISTICS OF CAREGIVERS, BRFSS, 2015-2017

**DOI:** 10.1093/geroni/igz038.673

**Published:** 2019-11-08

**Authors:** Valerie J Edwards, Lisa C McGuire, Erin D Bouldin, Christopher A Taylor

**Affiliations:** 1 Centers for Disease Control and Prevention, Atlanta, GA, Antigua and Barbuda; 2 Alzheimer's Disease and healthy aging program, centers for disease control & prevention, atlanta, Georgia, United States; 3 Appalachian State University, Boone, North Carolina, United States

## Abstract

As caregiving is becoming a leading public health issue, we used the BRFSS to better understand who caregivers are as well as features of their caregiving experience. We combined BRFSS data from three years (2015-2017) to examine demographic characteristics including gender, race, age, education, employment, and marital status. Slightly more than one in five BRFSS respondents (20.7%) were caregivers. The majority were women, had at least some college, were employed, and married or living with a partner. Most caregivers were under 45 years old, but a substantial proportion (20.7%) were 65 years or older. There was significant state variation by every demographic characteristic. These data underscore that caregiving is a common feature of U.S. family life, and caregivers’ concerns should be considered in healthcare, employment, and service provision. Important state-level variations uncovered by use of the BRFSS Caregiving Module could lead to better targeting of caregiver support programs.

